# Malaria and Pregnancy

**Published:** 1902-09

**Authors:** A. B. Greiner

**Affiliations:** Richmond, Va.


### MALARIA AND PREGNANCY.*

By A. B. GREINER, M.D.,

Richmond, Va.

Malarial fever needs no introduction to you; nor, I dare say, do you care about meeting it when it persists in making itself known in a pernicious way. Malaria is essentially a pathologic condition and one that proves some time to be a very stubborn one if we are to consider and believe that the mortality attendant upon

*Read before the Richmond Academy of Medicine and Surgery, May 27, 1902.

it, in some malarial regions, is quite appalling. Pregnancy, on the other hand, we may consider a physiological process, one that needs no lengthy treatise to establish it as such; and when not complicated by some pathologic condition runs a well-defined physiological course and terminates in a physiological way. We would then consider malaria and pregnancy antagonistic to each other; not only in a definitive way, but also because they run an essentially different course and end in a far different way. With the light of modern investigation into the cause, morbid anatomy, course and treatment of diseases shining in full force upon the result of our researches, we are compelled to dissent from an opinion formerly expressed, that "the treatment of intermittent fever in pregnancy is the same, so far as antiperiodics are concerned, as if the patient were not pregnant; and there need be no hesitation in giving quinine, for example, and giving it freely, unless some idiosyncrasy forbids its use."

From the statistics of abortion in malarial countries, it is conclusively shown that abortion occurs more frequently than in non-malarial countries, and we have therefore to accept the fact that intermittent fever in pregnant women predisposes to abortion and premature labor, consequently these two processes have been referred to as antagonistic. This antagonism manifests itself on the part of malaria by tending to abortion and premature labor; and on the part of pregnancy by conferring upon the woman a lessened tendency to contract malarial fever. How it is, if it is really the case, that pregnancy renders one less liable to malarial infection, may perhaps be explained by the fact that the pregnant woman is not so much exposed to those factors which at one time or another have been recognized as causes of intermittent fever. The woman, on account of her sedentary life indoors, is not exposed to so great an extent to the contaminating air, if we are to regard malaria as a true miasmatic disease; nor to the infecting anopheles, if it is the only true cause. The cases of malaria complicating pregnancy which I have seen ran about the same course as to the recurrence of the chill as do the cases of uncomplicated intermittent fever. Some observers claim that the fever comes to be of a remittent instead of an intermittent type in cases where these two conditions are associated. The specific antimalarial remedy, which has been for so long a time administered, is practically of no antipyretic value in conditions of fever due to any other cause, unless given in exceedingly large doses; and as this drug is so satisfactory in the successful treatment of malaria, it is the one of which we first think when seeking a remedy to be given, even in cases complicated by pregnancy, for it does prevent the chills and abate the fever. Those who are in position to speak authoritatively concerning the physiologic action of quinine, say that it is used for its stimulant effect on the uterus in cases of uterine inertia, because it does stimulate the uterus to more violent contractions. The opinion has been expressed that the drug does not initiate uterine contraction, but stimulates after contraction has begun. I am inclined to the belief that quinine does, in some cases, initiate contractions in the pregnant uterus; and this conclusion I have reached after observing that in some cases of pregnancy abortion was very promptly produced in women who were only a few weeks pregnant, and in whom there was no other ascertainable cause for the beginning of such contraction.

The predisposition to abortion observed in some women we might regard as a probable cause of initiatory contraction, yet why should this factor become operative after the administration of quinine if the quinine had nothing to do with it? Some explanation other than mere coincidence must be given for this. A recent writer has advanced the theory that the malaria is the potent cause of the abortion. While I regard the disease as a predisposing cause, yet I cannot understand why abortion should be delayed until the dose of quinine has been administered. Just how this drug exercises such stimulant effect on the uterus I am not prepared to explain, unless we are content to say it stimulates involuntary muscle fiber. The women who seem most prone to abort after an antimalarial dose of quinine are those who have previously done so, and in whom the various causes of abortion are apt to be successful.

I have seen one case in which abortion followed within twenty-four hours the administration of a ten-gain dose of quinine. Some writers say that pregnancy predisposes to an outbreak of chills and fever in those who have previously had such, and in whom at the time of impregnation the malarial poison is quiescent. But, whatever be the cause of malarial fever and the tendency for pregnant women to contract it, it is quite often met with and must be treated; treated in a very cautious manner if we are to leave undisturbed the pregnancy. It has been advised that one of the bromides be administered along with some salt of quinine in the treatment, the object of the bromide being clearly to depress the nerve centers that have to do with stimulation of the uterus. Others have advised black haw for the same effect. This plan of treatment is frequently carried out successfully, and with absolute quiet on the part of the patient nothing more can be desired.

The object of this article is not so much the recounting of old methods of treatment in the management of such cases, but while offering nothing new to emphasize the efficiency of a comparatively new drug which has come forward prominently, and justly so, in the treatment of malaria when pregnancy complicates. A few people have an idiosyncrasy to quinine and therefore cannot take it in quantity sufficient to combat the malarial toxins. Undoubtedly, in some cases pregnancy is a known idiosyncrasy and one that should be kept in mind. It has been the experience of some practitioners that quinine administered for its stimulant effect to the uterus during labor, predisposes to postpartum hemorrhage. This is a complication for which every one of us has a natural dread, and if it does cause such hemorrhage, it is our duty to discard it or give it cautiously and be alive to the possible result. It is not my purpose to underestimate the value of quinine, which has an established reputation as a specific in intermittent fever. Some other drugs have been used for their antiperiodic effect, but none so successfully as quinine.

The desire then, is for some agent that is sufficiently powerful as an antiperiodic and one that will not disturb gestation. No physician would allow his patient to suffer unnecessarily from chills and fever, even though she were pregnant, without giving her some control or some curative remedy, although there would be danger of interruption of the pregnancy. The disease is in itself an evil, and abortion from the use of drugs is not a good thing, and we are often forced to decide which would prove to be most disastrous. As a curative agent for intermittent fever methylene blue, within the last few years, has been advised; and while not so powerful as quinine in an antiperiodic way, yet undoubtedly has been proved to be very useful. This drug destroys the plasmodium malariæ and seems to be about as efficient as quinine in this particular, judging from clinical results, but this I have not determined from blood examinations. It had better be administered several hours before the time for the paroxysm and continued some hours afterward, or until no organisms are to be found in the blood. The only objection to its use, so far as I have been able to judge, is its intense irritant effect on the stomach if given in doses exceeding three grains. Of course we may look for prompt and complete evacuation of the stomach, but there is no disagreeable after effect. We may consider the dose to be from one to five grains, the maximum to be given to those who are not so susceptible to stomachic irritants, or to be given after meals. Methylene blue is eliminated by the kidneys and the urine is colored an intense blue. I have made use of this drug in cases of malaria not complicated by pregnancy, and in those people who do not seem to be greatly benefited by quinine, and while my experience in this particular has not been extensive, yet there were good results attending its use. Without entering into detail as to individual cases of malaria and pregnancy and the treatment thereof by this remedy, suffice it to say that in the few cases which I have treated the drug has been a very satisfactory one and it is recommended for your consideration.

*506 Grace Street, East.*

